# “Spin” in wound care research: the reporting and interpretation of randomized controlled trials with statistically non-significant primary outcome results or unspecified primary outcomes

**DOI:** 10.1186/1745-6215-14-371

**Published:** 2013-11-06

**Authors:** Suzanne Lockyer, Rob Hodgson, Jo C Dumville, Nicky Cullum

**Affiliations:** 1 Department of Health Sciences, University of York, Seebohm Rowntree Building, Heslington YO10 5DD, UK; 2 School of Nursing, Midwifery & Social Work, University of Manchester, Jean McFarlane Building, Oxford Road, Manchester M13 9PL, UK

**Keywords:** Wound care, Randomized controlled trials, Bias, Industry funding

## Abstract

**Background:**

Spin in the reporting of randomized controlled trials, where authors report research in a way that potentially misrepresents results and mislead readers, has been demonstrated in the broader medical literature. We investigated spin in wound care trials with (a) no statistically significant result for the primary outcome and (b) no clearly specified primary outcome.

**Methods:**

We searched the Cochrane Wounds Group Specialised Register of Trials for randomized controlled trials (RCTs). Eligible studies were: Parallel-group RCTs of interventions for foot, leg or pressure ulcers published in 2004 to 2009 (inclusive) with either a clearly identified primary outcome for which there was a statistically non-significant result (Cohort A) or studies that had no clear primary outcome (Cohort B).

We extracted general study details. For both Cohorts A and B we then assessed for the presence of spin. For Cohort A we used a pre-defined process to assess reports for spin. For Cohort B we aimed to assess spin by recording the number of positive treatment effect claims made. We also compared the number of statistically significant and non-significant results reported in the main text and the abstract looking specifically for spin in the form of selective outcome reporting.

**Results:**

Of the 71 eligible studies, 28 were eligible for Cohort A; of these, 71% (20/28) contained spin. Cohort B contained 43 studies; of these, 86% (37/43) had abstracts that claimed a favorable treatment claim. Whilst 74% (32/43) of main text results in Cohort B included at least one statistically non-significant result, this was not reflected in the abstract where only 28% contained (12/43) at least one statistically non-significant result.

**Conclusions:**

Spin is a frequent phenomenon in reports of RCTs of wound treatments. Studies without statistically significant results for the primary outcome used spin in 71% of cases. Furthermore, 33% (43/132) of reports of wound RCTs did not specify a primary outcome and there was evidence of spin and selective outcome reporting in the abstracts of these. Readers should be wary of only reading the abstracts of reports of RCTs of wound treatments since they are frequently misleading regarding treatment effects.

## Background

Randomized controlled trials (RCTs) are the best design for assessing the relative effectiveness of interventions in healthcare because, if well conducted, they provide unbiased estimates of treatment effects. However, even where RCTs are well conducted the way in which a trial is reported is also important. Studies focusing on reporting have mainly considered methodological issues such as the reporting of allocation concealment and blinding [1,2]. Recently, however, there has also been a focus on “spin” in trial reporting, whereby authors’ use of language and emphasis on results for particular outcomes potentially misleads readers [3-7]. As Boutron and colleagues [5] describe it, spin may “result from ignorance … unconscious bias, or wilful intent to deceive”. Whilst the concept of spin has been discussed in the *British Medical Journal* as far back as 1995 [8-11], and there have been a number of methodological reviews evaluating misleading claims in published reports of either RCTs [12-14] or systematic reviews [15], there has been little research into spin *per se*.

Boutron and colleagues [5] recently developed a method for identifying and classifying spin in RCT reports. They applied their approach to a cohort of medical journal published papers that reported statistically non-significant differences for the primary outcome. The authors reported that spin was present in the main text of 61% (44/72) papers and 68% (49/72) of abstracts. A number of further studies have observed spin in trial reports. Vera-Badillo and colleagues [6], focusing on clinical trials for women with breast cancer, reported that 59% (54/92) of studies that found a statistically non-significant difference for the primary outcome result contained spin in either the abstract or concluding statement, while Vedula and colleagues [7] observed that 66% (8/12) trials of gabapentin contained spin as well as providing a detailed account of how such spin was used in the promotion of gabapentin for off-label purposes.

We were interested in assessing the amount, type and level of spin in RCTs of treatments for wounds where most interventions are devices (rather than drugs) for which effectiveness data are not required for licensing and use in Europe [16]. In this current study we aimed to assess the prevalence of spin using the classification of Boutron and colleagues [5] in a cohort of wound care trials that reported no statistically significant difference. We have previously drawn attention to the misleading way in which research is referred to in wound product marketing literature [17]. As an extension of Boutron and colleagues [5] previous work, we also assessed the prevalence of wound RCTs that did not clearly specify the primary outcome in the trial report (to which the full application of the methodology described by Boutron and colleagues [5] is not possible).

Specification of primary outcomes is required by CONSORT (Consolidated Standards of Reporting Trials) [1] and non-specification may be related to outcome reporting bias (where outcomes are selected for report on the basis of statistical significance). This phenomenon has not been previously assessed in wounds research though it is not uncommon in other areas [2]. We anticipated non-specification of a primary outcome may be common in wounds research since there is a range of different outcome measures used including different measures of wound healing (for example, reduction in size versus complete healing) and no agreed core outcomes [18,19].

Where trialists report multiple outcomes without defining a primary outcome, we sought to test the hypothesis that ‘spin’ resulting from selective outcome reporting might be present – that is, the ‘cherry-picking’ of particular results to which extra emphasis is added within the study report (for example, over-emphasis on positive treatment effects). In particular, we sought to investigate whether statistically significant outcomes might be more frequently presented in abstracts to the exclusion of non-significant outcomes.

## Methods

### Eligibility, search and study selection

Eligible studies were: randomized evaluations of any interventions for treatment of foot, leg or pressure ulcers; reports published between 2004 and 2009, inclusive; English language only (because of lack of translation resources); and studies with a clearly specified primary outcome with a statistically non-significant difference for this treatment effect (defined as *P* = >0.05; classified as Cohort A) or studies with no primary outcome specified independent of its statistical significance (classified as Cohort B).

We confined our study to RCTs of leg, foot and pressure ulcer treatments since these are the most common types of chronic wound and there is an identifiable research community to whom the findings will be meaningful and relevant. The 5-year window of trial publications was selected in order to manage limited resources and the years 2004 to 2009 chosen as 2004 is 10 years after the first paper on trial reporting quality and therefore there had been sufficient time for reporting quality to improve [20].

Studies reported were considered to have specified a primary outcome where they explicitly defined a primary outcome in the introduction or methods section; reported use of an outcome in a power calculation; or where only a single outcome was reported. Studies with multiple primary outcomes were considered not to have identified a primary outcome and were included in Cohort B. Following Boutron and colleagues [5], only studies with a statistically non-significant result for a clearly specified primary outcome were included because the interpretation of these results is more likely to be subject to prior beliefs of effectiveness, resulting in biased interpretations [5]. Phase 1 trials and equivalence/non-inferiority trials were excluded since the aim of the former is not to test effectiveness and in the latter *P* values are not interpretable in the same way as a superiority trial. Trials described as pilot studies were also excluded if their objective was clearly to investigate the feasibility of a full trial, as were all conference abstracts. Studies were also excluded if they were secondary reports where the primary paper or main study report was referenced, or where it was clear it was a protocol or economic evaluation.

Studies were identified by searching the Cochrane Wounds Group Specialised Register of Trials (Cochrane Wounds Group resource). The register is maintained by the Cochrane Wounds Group, York, and aims to identify all randomized and quasi-randomized controlled trials in the area of wounds research. Reports are identified for inclusion in the register by regular searches of a number of databases including Medline, Embase, CINAHL and Central along with periodic searches of other databases. Studies included in the register have been coded on several criteria including wound type. A search was therefore carried out on publications in 2004 to 2009 (inclusive) using the following search terms in the condition field: Pressure* or Venous or Leg* or Ulcer* or Diabet*, and in the intervention field: Treat*.

The titles and abstracts (where available) of identified studies were screened by a single author (JCD) to exclude obviously irrelevant studies, based on the above eligibility criteria. The full text of the remaining papers was screened by two authors (RH and SL) after extensive piloting of the screening criteria and extraction form. Any disagreements were resolved through discussion and arbitrated by a third author (JCD) where agreement could not be reached.

### Data extraction

#### General

The development of the data extraction sheet was a process of iteration involving discussion between the three reviewers (JCD, RH, SL), with piloting at each stage.

For both Cohorts A and B the following general characteristics were extracted: wound type; number of trial arms; intervention and comparator(s); duration of follow-up; and funding source.

Data extraction was recorded on a Microsoft Excel spreadsheet using drop-down menus where appropriate. Data extraction was completed independently by two reviewers (SL, RH), with disagreements resolved through discussion and involvement of a third reviewer (JCD) where required. Kappa statistics were calculated.

#### Cohort A: studies with a statistically non-significant difference for the primary outcome result

This part of the study followed the methodology of Boutron and colleagues [5]. We applied the spin classification scheme to the following sections of each study in Cohort A: abstract results and conclusions sections; and main text results, discussion and conclusions sections (where there was no clear conclusion section the last paragraph that summarized the results was used).

In addition to identifying and classifying spin *per se*, we also assessed the level of spin in the abstract and main text conclusions according to Boutron and colleagues [5]. Boutron and colleagues [5] employed three schemes, one applied to the results sections (abstract and main text); one applied to the discussion section; and one applied to the conclusions sections (abstract and main text). These are as follows:

Results

•Focus on statistically significant within-group comparison

•Focus on significant secondary outcomes

•Focus on significant subgroup

•Focus on significant modified population (for example, per protocol)

•Focus on statistically significant within- and between-group comparisons of secondary outcomes

•Anything that at the discretion of the authors was considered to be spin and is not covered by the above categories

Discussion

•Focus on statistically significant within-group comparison

•Focus on significant secondary outcomes

•Focus on significant subgroup

•Focus on significant modified population (for example, per protocol)

•Claims equivalence

•Rules out adverse effect

Conclusion

•Claims effectiveness with no acknowledgement of non-significant results for primary outcome

•Claims equivalence

•Rules out adverse effect

•Acknowledges non-significance, but emphasizes significant results for other outcomes

•Acknowledges non-significance, but emphasizes treatment benefit

•Emphasizes benefit based on new outcome

•Anything that at the discretion of the authors was considered to be spin and is not covered by the above categories

The level of spin was classified as high, moderate, low or none according to the following criteria, with none acting as a default category:

•High spin: no acknowledgement of non-significant results for primary outcome and no uncertainty in framing and no recommendations for further trials

•Moderate spin: no acknowledgement of non-significant result for primary outcome and uncertainty in framing or recommendations for further trials

•Low spin: acknowledgement of non-significant results for primary outcome, but uncertainty in framing and recommendations for further trials

#### Cohort B: studies with no clearly defined primary outcome

Within Cohort B we counted the number of outcomes reported in the main text and abstract and then extracted the statistical significance of findings for each outcome and classified them as: significant (using *P* < 0.05) or not statistically significant (*P* ≥ 0.05). Where no statistical testing was conducted or clearly reported the statistical significance of the treatment effect was recorded as unclear. In addition, we recorded whether the abstract claimed an effect (that is, a positive claim about a treatment effect).

### Data analysis

Results were initially recorded in Microsoft Excel, and SPSS (Released 2009. PASW Statistics for Windows, Version 18.0. Chicago: SPSS Inc.) [21] was used for data analysis.

#### Cohort A: studies with statistically non- significant differences for the primary outcome

Descriptive summary statistics (number and percentage for categorical data; median, range and inter-quartile range for continuous data) were calculated for: the general characteristics of included studies (nature of funder, wound type, duration of follow up, intervention and comparator(s)); stratified by outcome type (continuous versus dichotomous), the prevalence of spin overall, and by section of the paper (that is, abstract, main results, main discussion and main conclusion); the types of spin used in each section of the paper; and the level of spin in the abstract and main text conclusions.

#### Cohort B: studies with no specified primary outcome

Descriptive summary statistics were calculated for: the general characteristics of included studies (number and percentage for categorical; median, range and interquartile range for continuous); summary of outcomes reported in the results section of the main text and the abstract, with comparison of the proportion of statistically significant findings in each; and number (proportion) of studies claiming a treatment effect in the abstract.

## Results

**Figure 1:**
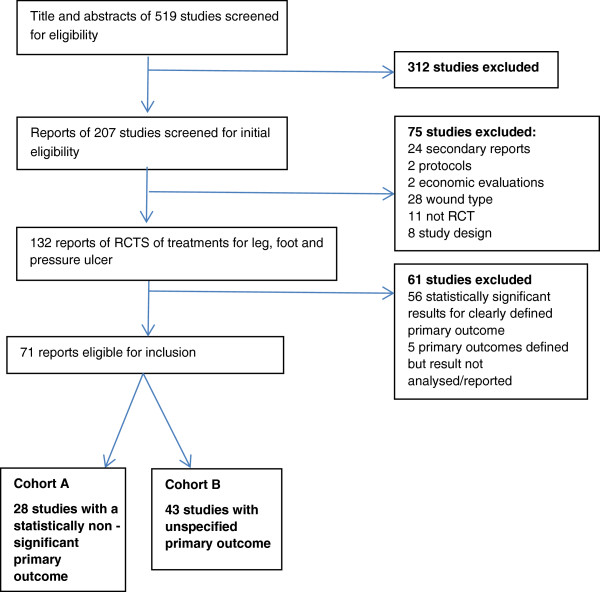
Review summary.

Of the 207 original study reports, 132 were primary reports of RCTs of interventions for leg, foot and pressure ulcers of which 71 met our inclusion criteria (Figure 1) (Additional file 1) – all study reports described only one relevant RCT. Twenty-eight studies reported a statistically non-significant result for the primary outcome and were analyzed for presence of spin (Cohort A) and 43 studies did not specify a primary outcome (Cohort B).

### Cohort A: studies with a statistically non-significant difference for the primary outcome

Agreement with regards to the extraction was fair to good, with Kappa statistics of 0.62, 0.74, 0.65, 0.75 and 0.76, respectively, for each of the following sections: abstract discussion, abstract conclusions, main report results, main report discussion and main report conclusions. Recourse to a third reviewer was required only three times. Table 1 summarizes the characteristics of the studies in Cohort A.

**Table 1:** Cohort A: summary of study characteristics

**Characteristics**	**Number**		**(%)**
	**n = 28**		
**Funding**			
Not for profit	10		(35.7)
For profit	9		(32.1)
Mixed (not for profit and for profit)	4		(14.3)
Not reported	3		(10.7)
Unclear	2		(7.1)
**Type of Wound**			
Leg ulcer	11		(39.3)
Diabetic foot ulcer	10		(35.7)
Pressure ulcer	6		(21.4)
Mixed ulcers	1		(3.6)
**Intervention**			
Device	13		(46.4)
Drug	10		(35.7)
Surgery	3		(10.7)
Care management	2		(7.1)
**Comparator**			
Placebo	7		(25.0)
Usual care	5		(17.9)
Device	12		(42.9)
Drug	2		(7.1)
Surgery	1		(3.6)
Care management	1		(3.6)
**Duration of follow-up**	**Median**	**(IQR)**	**Range**
Weeks	12	[8-23]	(3–156)

### Cohort A: prevalence and type of spin in the abstract and main text

In total, 63% (17/27) of abstracts contained spin (one study had no abstract) with 30% (8/27) of abstracts claiming effectiveness of the intervention. A further 22% of abstracts (6/27) acknowledged the non-significant result for the primary outcome, but emphasized other significant results. Over half of the abstract conclusions contained spin (59%; 16/27).

Nearly three-quarters (71%) of reports contained spin in the main text (that is, spin in any category; Table 2). The prevalence of spin was highest in the main text conclusions (61%; 17/28) where the most common forms of spin were claims of equivalence or effectiveness without acknowledging that there was no statistically significant difference for the primary outcome. In some cases, multiple types of spin were used. We also observed that emphasis on secondary outcomes both within the results and discussion section was only adopted when the primary outcome was a continuous one.

**Table 2:** Cohort A: prevalence and type of spin by main text by section

	**Dichotomous outcomes**		**Continuous outcomes**		**All studies**	
	**Number**	**(%)**	**Number**	**(%)**	**Number**	**(%)**
	**n = 13**		**n = 15**		**n = 28**	
Results – any type*	4	(30.8)	4	(26.7)	8	(28.6)
Focus on statistically significant results from:						
Within-group analyses	0	(0.0)	0	(0.0)	0	(0.0)
Subgroups	4	(30.8)	3	(20.0)	7	(25.0)
Secondary outcomes	0	(0.0)	3	(20.0)	3	(10.7)
Per protocol analysis	2	(15.4)	1	(6.7)	3	(10.7)
Other	1	(7.7)	0	(0.0)	1	(3.6)
**Discussion – any type**	8	(61.5)	8	(53.3)	16	(57.1)
Focus on statistically significant results from:						
Within-group analyses	0	(0.0)	0	(0.0)	0	(0.0)
Subgroups	4	(30.8)	4	(26.7)	8	(28.6)
Secondary outcomes	0	(0.0)	5	(33.3)	5	(17.9)
Per protocol analysis	2	(15.4)	1	(6.7)	3	(10.7)
Claims equivalence	0	(0.0)	0	(0.0)	0	(0.0)
Rules out adverse effect	1	(7.7)	5	(33.3)	6	(21.4)
Other	5	(38.5)	0	(0.0)	5	(17.9)
**Conclusions – any type**	7	(53.8)	10	(66.6)	17	(60.7)
Claims effectiveness with no acknowledgement of NS results for primary outcome	4	(30.8)	4	(26.7)	8	(28.6)
Claims equivalence	1	(7.7)	5	(33.3)	6	(21.4)
Rules out adverse effect	0	(0.0)	0	(0.0)	0	(0.0)
Acknowledges non-significance, but emphasizes significant results for other outcomes	3	(23.1)	4	(26.7)	7	(25.0)
Acknowledges non-significance, but emphasizes treatment benefit	2	(15.4)	3	(20.0)	5	(17.9)
Emphasizes benefit based on new outcome	1	(7.7)	3	(20.0	4	(14.3)
Other	0	(0.0)	0	(0.0)	2	(7.1)

*More than one type could be used in each section of a report. NS, non-significant.

### Cohort A: level of spin

The overall prevalence of spin in the abstract and main text conclusions was comparable (59% compared with 61%, respectively; Table 3). However, nearly 22% (6/27) of abstract conclusions had a high level of spin compared with 11% (3/28) of main text conclusions. We noted that in the main text conclusions, authors were more likely to acknowledge the non-significance of results and/or recommend further trials, thus reducing the level of spin.

**Table 3:** Cohort A: level of spin in abstract and main text conclusion sections

**Level**	**Number(%)**			**Number(%)**				
	**Abstract conclusions**		**Main text conclusions**					
	**n = 27**		**n = 28**					
**None**	11	(40.7)	11	(39.3)
**Low**	4	(14.8)	4	(14.3)
**Moderate**	6	(22.0)	10	(35.7)
**High**	6	(22.0)	3	(10.7)

### Cohort A: prevalence of spin by funding type

The source of funding could be determined for 82% of trials (23/28; Table 4) with an even distribution of for profit and not for profit funding (36%; 10/28 and 36%; 10/28, respectively).

**Table 4:** Cohort A: prevalence of spin by funding source

**Funding source**	**Number(%)**		**Number(%)**	
	**Spin in abstract conclusions**		**Spin in main text conclusions**	
	**n = 27**		**n = 28**	
**For profit (abstract n = 9; main n =10)**	8	(88.9)	9	(90.0)
**Mixed (n = 4)**	1	(25.0)	2	(50.0)
**Not for profit (n = 10)**	6	(60.0)	5	(50.0)
**Unclear/Not reported (n = 4)**	1	(25.0)	1	(25.0)
**Total**	17	(63.0)	16	(57.1)

Table 4 shows whether there was spin in the main text and abstract conclusions, stratified by funding source. Originally, inferential analysis was planned to investigate the influence of funding, but due to the small sample size it was decided to present only a descriptive analysis. In total, 89% (8/9) of the industry funded trials had some element of spin in the abstract conclusions and 90% (9/10) had some element of spin in the main text conclusions. This was somewhat higher than the prevalence of spin observed in not-for-profit funded studies where 60% (6/10) had some element of spin in the abstract and 50% (5/10) had spin in the main text. However, only 25% (1/4) of reports of trials with mixed funding (that is both for profit and not for profit) had some element of spin in the abstract conclusions and 50% (2/4) in the main text conclusions.

### Cohort B: studies with no specified primary outcome

Cohort B comprised 43 studies representing 33% (43/132) of identified RCTs of treatments for leg, foot and pressure ulcers published between 2004 and 2009 in English language journals (Table 5) (Additional file 1).

**Table 5:** Cohort B: summary of study characteristics

**Characteristics**	**Number**		**(%)**
	**n = 43**		
**Funding**			
Not for profit	10		(23.3)
For profit	11		(25.6)
Mixed (not for profit and for profit)	2		(4.7)
Not reported	20		(45.6)
Unclear	0		(0.0)
**Type of Wound**			
Leg ulcer	24		(55.8)
Diabetic foot ulcer	13		(30.2)
Pressure ulcer	5		(11.6)
Mixed ulcers	1		(2.3)
**Intervention**			
Device	28		(65.1)
Drug	12		(27.9)
Surgery	1		(2.3)
Care management	2		(4.7)
**Comparator (note, in trials >2 arms there is >1 comparator)**			**n = 51**
Placebo	19		(37.3)
Usual care	8		(15.7)
Device	17		(33.3)
Drug	4		(7.8)
Surgery	2		(3.9)
Care management	1		(2.0)
**Duration of follow up**	**Median**	**(IQR)**	**Range**
Weeks	8	(4–13)	2–104

In the 43 studies comprising Cohort B, a median of 9 individual outcomes (IQR 6–16) were reported in the main text results and a median of 3 (IQR 2–5) in the abstract results, compared to Cohort A where there were a median of 4 (IQR 2–7) outcomes reported in the main text results and a median of 2 (IQR 2–4) in the abstract results.

### Cohort B: influence of statistical significance on reporting in the absence of a specified primary outcome

In total, 72% (31/43) of main text results in Cohort B included at least one statistically significant result and 74% (32/43) at least one statistically non-significant outcome (Table 6). Fewer abstracts contained at least one statistically significant result (51%; 22/43); however, only 28% of abstracts (12/43) contained at least one statistically non-statistically significant outcome.

**Table 6:** Cohort B: comparison between the statistical significance of results presented in the main text results section and abstract results section of reports with unclear primary outcome

	**Corresponding abstract results; number containing:**			
**Main text results; number of reports containing:**	**Outcomes with no reference to statistical significance (n =16 including 3 with no abstract)**	**SS only (n = 15)**	**SNS only (n = 5)**	**SS and SNS (n = 7)**
**Row A: Outcomes with no reference to statistical significance (n = 6)**	100% (6)	0% (0)	0% (0)	0% (0)
**Row B: SS only (n = 5)**	0% (0)	100% (5)	0% (0)	0% (0)
**Row C: SNS only (n = 6)**	33% (2)	0% (0)	50% (3)	17% (1)
**Row D: SS and SNS (n = 26)**	31% (8)	39% (10)	8% (2)	23% (6)

SNS, statistically non-significant; SS statistically significant.

### Cohort B: proportion of studies claiming a treatment effect in the abstract conclusions

In Cohort B, 86% (37/43) of abstracts claimed a favorable treatment effect (Table 7). Of these abstracts, 41% (15/37) presented either no statistical analysis or only statistically non-significant findings in support of the claim. A further 41% (15/37) of the abstracts presented only statistically significant results, even though many of these trials presented both statistically significant and non-significant findings in the main text (Table 6).

**Table 7:** Cohort B: use of statistical testing to support claims of effectiveness in trial reports

	**Abstract results section**			
**Claim effectiveness in abstract?**	**No reference to statistical significance (n =16 including 3 with no abstract)**	**SNS only (n = 5)**	**SS only (n = 15)**	**SS and SNS (n = 7)**
**Yes (n = 37; 86%)**	32% (12)	8% (3)	41% (15)	19% (7)
**No (n = 3; 7%)**	33% (1)	67% (2)	0% (0)	0% (0)
**No Abstract (n = 3; 7%)**	100% (3)	0% (0)	0% (0)	0% (0)

SNS, statistically non-significant; SS, statistically significant.

## Discussion

This study is the first to appraise the prevalence of spin in reports of trials of interventions for foot, leg or pressure ulcers. A key finding was that a third of the wound trial reports did not specify a primary outcome, a figure comparable with that reported in the general medical literature [5].

### Key findings for studies with a statistically non-significant difference for the primary outcome result (Cohort A)

We found that, as in general medical journals, spin is commonly employed in reports of wound trials where there is no statistically significant result for the primary outcome. We found the lowest prevalence of spin in the results sections of the main text and the highest in discussion and conclusion sections. Boutron and colleagues [5] reported that 33% of general medical journal abstracts contained a high level of spin in the conclusions, whilst we report a prevalence of 22% with 59% of abstract conclusions having some level of spin. We identified spin in reports of wound research funded by both for-profit and not-for-profit agencies but did not have a large enough sample to determine whether a significant association with type of funding is associated with spin. Further conclusions regarding the role of the funder are therefore not possible on the basis of this study.

### Key findings for studies with no specified primary outcome (Cohort B)

Where studies did not clearly specify a primary outcome, there is potential for spin in the form of emphasizing study results based on the results of significance testing rather than the importance of the outcome. Our findings suggest that such ‘cherry picking’ of statistically significant results is common place in wound care trials with no clear primary outcome. We found a discontinuity in the proportions of statistically significant and non-significant results reported between main texts and their corresponding abstracts. Whilst in the main text nearly three-quarters of reports included at least one statistically non-significant result, only a quarter of abstracts contained at least one non-significant result. Furthermore, whereas statistically significant results from the main text results always appear in the abstract, only a third of non-significant results did so. This seems to provide evidence of selective presentation of statistically significant findings in study abstracts.

### Importance of findings

Recent work on outcome reporting bias has largely focused on the selective reporting of a subset of outcomes from the full set measured; in other words, a discrepancy between study protocols and reports [3,4]. Other studies have shown how high proportions of trial abstracts and main text fail to meet international reporting standards such as CONSORT (Consolidated Standards of Reporting Trials), especially with respect to the reporting of methodological information [22]. Our study lends further support to the notion that spin is a major issue, with abstracts being particularly prone to distorted presentation of findings. Where there is no clear primary outcome specified, there is also evidence that distortion of findings in the main text emphasizes the benefits of treatments based on selected use of evidence. Such discordance between report sections of trials has also recently been noted when comparing abstract conclusions with main text conclusions — with stronger statements of support for treatments in the abstract [23].

Distorted reporting in abstracts is of particular concern as these sections are easily accessed (and freely available), so clinical decisions may be based on abstracts alone [24]. Even when the full report is available, readers may only scan the abstract and conclusions [12,25]. These issues are of particular relevance in wound care, an area of healthcare where many of the treatment decisions are made by nurses working in community settings, where there are significant time constraints and limited access to computers and research findings.

### Limitations

When determining whether the results of the included studies were statistically significant or not, we used *P* < 0.05/≥ 0.05 as the criterion. We recognize that, generally, trial outcomes should not be judged or interpreted solely on this cut-off. We note that our application of this cut-off reflects reporting practice within the included studies as well as general interpretation of statistical findings.

We also note that the first screening phase of this work (study titles and abstracts) was only conducted by one reviewer. Ideally this should have been undertaken by two reviewers but this was not possible at the time. The single reviewer was very experienced in conducting systematic reviews in the field and also took a conservative approach, meaning she included all the studies where there was doubt as to eligibility and these were retrieved as full text. Two reviewers were involved in the second stage sifting of the full text of studies, as highlighted by the large number of studies (approximately 100) that were excluded at that stage.

The methodology for Cohort A was based on a published classification of spin, but beyond Boutron and colleagues [5] there is no further guidance on its use, so the methods we adopted may have differed in practice. However, we found application of Boutron and colleagues [5] methods viable and using this previous work meant findings from this work could be related to a general cohort of trial reports. However, as noted by Boutron and colleagues [5], the assessment of spin has a subjective element. To minimize any negative effects of such subjectivity we undertook detailed preparatory work regarding the identification and classification of spin in order to try and harmonize the process. As such, agreement between the two independent reviewers was found to be fair to good. Due to incomplete reporting we were not able to classify the source of funding for a number of studies. It is likely that the non-reporting of funding is not random and may represent a potential source of bias. Finally, whilst the sample size for Cohort A was small, limiting possible analysis of factors which may influence spin, we note this does reflect all trial report evidence available to us over the period of interest.

The second part of the study (Cohort B) was exploratory; data extraction was therefore limited to the results and conclusions sections to identify the number of outcomes and the conclusions drawn. The study was based on outcomes reported in the results section only and trial protocols were not checked.

## Conclusions

We have demonstrated for the first time that a large proportion of the reports of interventions for foot, leg or pressure ulcers are affected by “spin”. We have also demonstrated that a high proportion of wound trials do not specify a primary outcome and these seem to selectively present outcomes, perhaps based on the results of significance testing.

Given the high proportion of papers that contain spin, end-users of wound care research need to be aware of the types of spin used and should approach papers with a clear sense of which outcomes are important to them (and particularly, patients). Readers should be wary of papers which do not clearly present important outcomes such as healing in both the results and conclusions. Readers should avoid relying on the abstract as a reliable report of a wound care trial. Critical appraisal skills training for all users of research is potentially beneficial as way of combating the impact of spin on clinical decision making. Investigators, editors and peer reviewers need awareness of the issue of spin and the importance of objectivity in research reporting. There is also scope for further research into both trial reports which do not define a primary outcome and in particular the impact on end-users.

## Abbreviations

RCT: randomized controlled trial.

## Competing interests

The authors declare that they have no competing interests.

## Authors’ contributions

The project was conceptualized and designed by JCD and NC. SL conducted the work as part of her Master’s Program. RH also conducted data screening and data extraction and conducted analysis for the paper. RH and JCD drafted the first version of the manuscript based on SL dissertation work and all authors have commented extensively and approved the final version. All authors read and approved the final manuscript.

## Supplementary Material

Additional file 1 Supplementary online content. Click here for file
