# Supplementary material for: “Spin” in wound care research: the reporting and interpretation of randomized controlled trials with statistically non-significant primary outcome results or unspecified primary outcomes
Source: Trials. 2013 Nov 6;14:371. doi: 10.1186/1745-6215-14-371 (PMC3832286; doi:10.1186/1745-6215-14-371)
Supplement: Additional file 1 — Supplementary online content. [file 1745-6215-14-371-S1.docx]

# Supplementary online content

**eTable 1.** Examples of Spin Strategies

**eTable 2.** Included studies in Cohort A

**eTable 3.** Included studies in Cohort B

**eTable 1.** Examples of Spin Strategies

| **Spin Strategy** | **Example** |
| --- | --- |
| **Title** |  |
| Claims treatment effectiveness. | “Integrating the results of phase IV (postmarketing) clinical trial with four previous trials reinforces the position that regranex (Becaplermin) gel 0.01% is an effective adjunct to the treatment of diabetic foot ulcers”[1] |
| Focusing on the treatment and the disease with no statements of a comparison or randomisation or no question about the effect of the treatment | “Apligraf in the treatment of neuropathic diabetic foot ulcers."[2] |
| **Results section** |  |
| Focus on statistically significant secondary outcomes. | “The primary endpoint, healing speed was similar in AQAg-dressed and CA-dressed wounds AQAg 0.29 ± 0.33 cm^2^ or 11.66 ± 17.7% per week; CA 0.26 ± 0.90 cm^2^ or 10.0 ± 15.5% per week; P=0.993; Table 2)…there were statically significant differences between groups in secondary end-points: ulcer depth reduction (fig.2)… Ulcer depth reduction… was twice as much in the AQAg group compared with CA dressings.”[3]  *Note: The statistically significant secondary outcome reduction in ulcer depth was the only secondary endpoint to be present in a figure.* |
| Focus on statistically significant subgroup analyses (ie, authors highlight a subgroup analysis that was not prespecified or was not adequately analyzed [no test of interaction and not analyzed on the  primary outcome]). | “In population with baseline PU area <=8cm2, mean absolute decrease in wound area at week 6 was significantly greater in the OKG group compared to placebo (-2.3 +/-4.2 cm2 vs -1.7+/- 1.7 cm2, p=0.006).” [4]  *Note: This is secondary outcome of a subgroup; PO (% change in area) is reported, but only for subgroups, not for whole group.* |
| Focuses on statistically significant outcomes from analysis of either per-protocol population or analyses of compliant patients only. | “…ninety one patients were included and followed up.... None of the patients was lost to follow up"... the results below concern only the patients included in the PP analysis: 77 patients who were totally concordant with the inclusion criteria.” [5]  *Note: All results are for the per protocol population, although unclear how arrived at; no flow chart.* |
| **Discussion section** |  |
| Reporting of statistically non-significant outcome as if the trial were an equivalence trial. | “It has shown that healing rates were similar when using Mepilex and Allevyn. It has shown that patients tolerated the Mepilex in a similar way to those on Allevyn with similar proportions withdrawing.” [6] |
| Focus on statistically significant secondary outcomes. | “Although the dressing groups did not differ in primary endpoints, [statistically] significantly greater depth reduction and overall wound bed improvement at final clinical evaluation were noted in the AQAg group…” [3] |
| Focus on statistically significant subgroup analyses (ie, no caution in the interpretation of the subgroup analyses results). | “As standardized care arm results improve, it is more difficult to show statistically different improvements with the addition of growth factors. Despite these caveats, there was evidence for preferential healing of target ulcers with baseline areas of less tn 1.46cm^2^ in subjects treated with Ragranex Gel 0.01% (P =0.0286).” [1] |
| Focuses on statistically significant outcomes from analysis of either per-protocol population or analyses of compliant patients only. | “In the PP population, twice-weekly treatment showed a dose-dependent 1 pr 10 µg of Chrysalin® increased the 20-week incidence of complete closure by approximately 45 and 72%, respectively, over the that observed in placebo…” [7] |
| **Conclusion section** |  |
| Conclusion claiming equivalence for statistically non-significant results. | “This trial showed that Urgotul and DuoDERM dressings, although different in nature were similar in terms of efficacy in the local management of leg ulcers.” [5] |
| Conclusion claiming efficacy with no consideration of the statistically non-significant results for the primary outcome. | “We conclude that the use of felt deflective padding represents a safe and effective method of pressure offloading...It can be applied rapidly without delays thus mitigating the need for more rigorous offloading in some patients.” [8] |
| Conclusion focusing on only statistically significant results (ie, secondary outcomes, subgroup analyses, within-group comparison, per protocol population analyses). | “This study suggested that the use of Apligraf resulted in a shorter time to wound healing (p=0.059) and a greater proportion of healed target ulcers (p=0.049) and was not associated with any product related serious adverse events. The overall results suggest that Apligraf, in combination with debridement, standard wound care and offloading, should be considered in treating patients with non-healing neuropathic diabetic foot ulcers.” [2] |
| Acknowledge statistically non-significant results for the primary outcome but with emphasis nonetheless on the beneficial treatment effect. | “This study supports the proposal that hydrogel dressing promotes epithelialisation. It also highlighted a trend towards faster healing in the treatment group, although this was not statistically significant. In addition, there were practical advantages for the hydrogel – shorter dressing times and less frequent changes.” [9]  *Note: PO was rate of healing which as non-significant (p=0.97); epithelialisation was a sub group; ease of use not specifically noted as an outcome.* |
| Acknowledge statistically non-significant results for the primary outcome but emphasis is on other statistically significant results (secondary outcomes, subgroup analyses, within-group comparison, per protocol population analyses). | “Overall while healing speed, the primary end-point, was comparable in the two groups, the AQAg dressed subjects experienced significantly greater depth reduction. There was a trend towards more overall wound improvement/less deterioration with AQAg dressed subjects, statistically significant in subjects prescribed antibiotics.” [3] |

**References**

1. Robson MC, Payne WG, Garner WL, Biundo J, Giacalone VF, Cooper DM: **Integrating the results of Phase IV (postmarketing) clinical trial with four previous trials reinforces the position that Regranex (Becaplermin) Gel 0.01% is an effective adjunct to the treatment of diabetic foot ulcers.** *The Journal of Applied Research* 2005, 5:**35-45.**
2. Edmonds M; European and Australian Apligraf Diabetic Foot Ulcer Study Group. (2009). **Apligraf in the treatment of neuropathic diabetic foot ulcers.** *The International Journal of Lower Extremity Wounds* 2009, 8:11-18.
3. Jude EB, Apelqvist J, Spraul M, Martini J; Silver Dressing Study Group: **Prospective randomized controlled study of Hydrofiber dressing containing ionic silver or calcium alginate dressings in non-ischaemic diabetic foot ulcers.** *Diabetic Medicine* 2007, **24:**280-288.
4. Meaume S, Kerihuel JC, Constans T, Teot L, Lerebours E, Kern J, Bourdel Marchasson I: **Efficacy and safety of ornithine alpha-ketoglutarate in heel pressure ulcers in elderly patients: results of a randomized controlled trial.** *The Journal of Nutrition*, Health and Aging 2009, 1**3:**623-629.
5. Meaume S, Ourabah Z, Cartier H, Granel-Brocard F, Combemale P, Bressieux JM, Bohbot S: **Evaluation of a lipidocolloid wound dressing in the local management of leg ulcers.** *Journal of Wound Care* 2005, **14:**329-334.
6. Franks PJ, Moody M, Moffatt CJ, Hiskett G, Gatto P, Davies C, Furlong WT, Barrow E, Thomas H; Wound Healing Nursing Research Group. **Randomized trial of two foam dressings in the management of chronic venous ulceration.** ***Wound Repair and Regeneration*** 2007, **15:**197-202.
7. Fife C, Mader JT, Stone J, Brill L, Satterfield K, Norfleet A, Zwernemann A, Ryaby JT, Carney DH.: **Thrombin peptide Chrysalin stimulates healing of diabetic foot ulcers in a placebo controlled phase I/II study.** *Wound Repair and Regeneration* 2007, **15:**23-34.
8. Nube VL, Molyneaux L, Bolton T, Clingan T, Palmer E, Dennis K:. **The use of felt deflective padding in the management of plantar hallux and forefoot ulcers in patients with diabetes.** *The Foot* 2006, **16:**38-43.
9. Kaya AZ, Turani N, Akyüz M: **The effectiveness of a hydrogel dressing compared with standard management of pressure ulcers.** *Journal of Wound Care* 2005, **14:** 42-44.

**etable 2.** Included studies in Cohort A

| **Study ID** | **Reference** |
| --- | --- |
| 5 | Nube VL, Molyneaux L, Bolton T, Clingan T, Palmer E, Dennis K:. **The use of felt deflective padding in the management of plantar hallux and forefoot ulcers in patients with diabetes.** *The Foot* 2006, **16:**38-43. |
| 9 | Meaume S, Ourabah Z, Cartier H, Granel-Brocard F, Combemale P, Bressieux JM, Bohbot S: **Evaluation of a lipidocolloid wound dressing in the local management of leg ulcers.** *Journal of Wound Care* 2005, **14:**329-334. |
| 30 | Taly AB, Sivaraman Nair KP, Murali T, John A: **Efficacy of multiwavelength light therapy in the treatment of pressure ulcers in subjects with disorders of the spinal cord: a randomized double-blind controlled trial.** Arch Phys Med Rehabil 2004, **85**: 1657-1661. |
| 31 | Harrison MB, Graham ID, Lorimer K, Vandenkerkhof E, Buchanan M, Wells PS, Brandys T, Pierscianowski T: **Nurse clinic versus home delivery of evidence-based community leg ulcer care: a randomized health services trial.** *BMC Health Services Research* 2008, **8:**243 |
| 42 | Leach MJ, Pincombe J, Foster G: **Clinical efficacy of horsechestnut seed extract in the treatment of venous ulceration.** *Journal of Wound Care* 2006, **15:**159 -167 |
| 43 | Franks PJ, Moody M, Moffatt CJ, Hiskett G, Gatto P, Davies C, Furlong WT, Barrow E, Thomas H; Wound Healing Nursing Research Group. **Randomized trial of two foam dressings in the management of chronic venous ulceration.** *Wound Repair and Regeneration* 2007, **15:**197-202. |
| 49 | Kopera D, Kokol R, Berger C, Haas J: **Does the use of low-level laser influence wound healing in chronic venous leg ulcers?** *Journal of Wound Care* 2005, **14:**391-394. |
| 78 | Dumville JC, Worthy G, Bland JM, Cullum N, Dowson C, Iglesias C, Mitchell JL, Nelson EA, Soares MO, Torgerson DJ; VenUS II team: Laval therapy for leg ulcers (VenUS II): randomised controlled trial. *BMJ* 2009 **338:**b773 |
| 81 | Driver VR, Hanft J, Fylling CP, Beriou JM: **Autologel Diabetic Foot Ulcer Study Group: A prospective, randomized, controlled trial of autologous platelet-rich plasma gel for the treatment of diabetic foot ulcers.** *Ostomy Wound Management* 2006, **52:**68-70 |
| 87 | Franks PJ, Moody M, Moffatt CJ, Martin R, Blewett R, Seymour E, Hildreth A, Hourican C, Collins J, Heron A; Wound Healing Nursing Research Group: **Randomized trial of cohesive short-stretch versus four-layer bandaging in the management of venous ulceration.** *Wound Repair and Regeneration* 2004 **12:**157-162 |
| 89 | Gupta A, Taly AB, Srivastava A, Kumar S, Thyloth M: **Efficacy of pulsed electromagnetic field therapy in healing of pressure ulcers: a randomized controlled trial.** *Neurology India* 2009 **57:**622-626. |
| 90 | van Gent WB, Hop WC, van Praag MC, Mackaay AJ, de Boer EM, Wittens CH. **Conservative versus surgical treatment of venous leg ulcers: a prospective, randomized, multicenter trial.** *Journal of Vascular Surgery* 2006, **44:**563-571. |
| 109 | Jude EB, Apelqvist J, Spraul M, Martini J; Silver Dressing Study Group: **Prospective randomized controlled study of Hydrofiber dressing containing ionic silver or calcium alginate dressings in non-ischaemic diabetic foot ulcers.** *Diabetic Medicine* 2007, **24:**280-288. |
| 112 | Kaya AZ, Turani N, Akyüz M: **The effectiveness of a hydrogel dressing compared with standard management of pressure ulcers.** *Journal of Wound Care* 2005, **14:** 42-44. |
| 128 | Meaume S, Kerihuel JC, Constans T, Teot L, Lerebours E, Kern J, Bourdel Marchasson I: **Efficacy and safety of ornithine alpha-ketoglutarate in heel pressure ulcers in elderly patients: results of a randomized controlled trial.** *The Journal of Nutrition*, *Health and Aging* 2009, 1**3:**623-629. |
| 136 | Piaggesi A, Macchiarini S, Rizzo L, Palumbo F, Tedeschi A, Nobili LA, Leporati E, Scire V, Teobaldi I, Del Prato S: **An off-the-shelf instant contact casting device for the management of diabetic foot ulcers.** *Diabetes Care* 2007, **30:**586-590. |
| 139 | Payne WG, Wright TE, Ochs D, Mannari RJ, Robson Martin: **The Dermagraft Pressure Ulcer Study Group: An exploratory study of dermal replacement therapy in the treatment of stage III pressure ulcers.** *The Journal of Applied Research* 2004, **4:**12-22. |
| 142 | Puttirutvong P: **Meshed skin graft versus split thickness skin graft in diabetic ulcer coverage.** *J Med Assoc Thai* 2004, **87:**66-72. |
| 146 | Robson MC, Payne WG, Garner WL, Biundo J, Giacalone VF, Cooper DM: **Integrating the results of Phase IV (postmarketing) clinical trial with four previous trials reinforces the position that Regranex (Becaplermin) Gel 0.01% is an effective adjunct to the treatment of diabetic foot ulcers.** *The Journal of Applied Research* 2005, **5:**35-45. |
| 169 | Van De Weg FB, Van Der Windt DA, Vahl AC: **Wound healing: total contact cast vs. custom-made temporary footwear for patients with diabetic foot ulceration.** *Prosthetics and Orthotics International* **32:**3-11. |
| 172 | Weed B, Davis MDP, Felty CL, Liedl DA, Pineda AA, Moore B, Rooke TW: **Autologous platelet lysate product versus placebo in patients with chronic leg ulcerations: a pilot study using a randomized double-blind, placebo-controlled trial.** *Wounds* 2004, **16:**273-282. |
| 174 | Zerón HM, Krötzsch Gómez FE, Muñoz RE: **Pressure ulcers: a pilot study for treatment with collagen polyvinylpyrrolidone.** *International Journal of Dermatology* 2007, **46:**314-317. |
| 176 | Jull A, Walker N, Parag V, Molan P, Rodgers A; Honey as Adjuvant Leg Ulcer Therapy trial collaborators: **Randomized clinical trial of honey-impregnated dressings for venous leg ulcers.** *British Journal of Surgery* **95:**175-182. |
| 181 | Edmonds M; European and Australian Apligraf Diabetic Foot Ulcer Study Group. (2009). **Apligraf in the treatment of neuropathic diabetic foot ulcers.** *The International Journal of Lower Extremity Wounds* 2009, 8:11-18. |
| 182 | Fernández-Montequín JI, Infante-Cristiá E, Valenzuela-Silva C, Franco-Pérez N, Savigne-Gutierrez W, Artaza-Sanz H, Morejón-Vega L, González-Benavides C, Eliseo-Musenden O, García-Iglesias E, Berlanga-Acosta J, Silva-Rodríguez R, Betancourt BY, López-Saura PA; Cuban Citoprot-P Study Group: **Intralesional injections of Citoprot-P (recombinant human epidermal growth factor) in advanced diabetic foot ulcers with risk of amputation.** *International Wound Journal* 2007, **4:**333-343. |
| 184 | Fife C, Mader JT, Stone J, Brill L, Satterfield K, Norfleet A, Zwernemann A, Ryaby JT, Carney DH.: **Thrombin peptide Chrysalin stimulates healing of diabetic foot ulcers in a placebo controlled phase I/II study.** *Wound Repair and Regeneration* 2007, **15:**23-34. |
| 187 | Landsman A, Roukis TS, DeFronzo DJ, Agnew P, Petranto RD, Surprenant M: **Living cells or collagen matrix: which is more beneficial in the treatment of diabetic foot ulcers?** *Wounds* 2008, **20:**111-116. |
| 189 | Michaels JA, Campbell B, King B, Palfreyman SJ, Shackley P, Stevenson M: **Randomized controlled trial and cost-effectiveness analysis of silver-donating antimicrobial dressings for venous leg ulcers (VULCAN trial).** *British Journal of Surgery* 2009, **96:**1147-1156. |

**eTable 3.** Included studies in Cohort B

| **Study ID** | **Reference** |
| --- | --- |
| 25 | Ferrara F, Meli F, Raimondi F, Amato C, Bonura F, Mulè G, Novo G, Novo SS: **The treatment of venous leg ulcers: a new therapeutic use of iloprost.** *Annals of Surgery* 2007, **246:**860-865. |
| 32 | Gethin G, Cowman S: **Manuka honey vs hydrogel - a prospective, open label, multicentre, randomised controlled trial to compare desloughing efficacy and healing outcomes in venous ulcers.** *Journal of Clinical Nursing* 2009, **18:**466-474. |
| 41 | Mariani F, Mattaliano, V, MOsti G, Gasbarro V, Bucalossi M, Blättler F, Amsler S: **The treatment of venous leg ulcers with a specifically designed compression stocking kit.** *Phlebologie* 2008*,* **37:**191-197 |
| 48 | Jünger M, Arnold A, Zuder D, Stahl HW, Heising S: **Local therapy and treatment costs of chronic, venous leg ulcers with electrical stimulation (Dermapulse): a prospective, placebo controlled, double blind trial.** *Wound Repair and Regeneration* 2008, **16:**480-487. |
| 51 | Franek A, Taradaj J, Polak A, Cierpka L, Blaszczak E: **Efficacy of high voltage stimulation for healing of venous leg ulcers in surgically and conservatively treated patients.** *Phlebologie* 2006, **35:**127-133. |
| 56 | Adunsky A, Ohry A; DDCT Group: **Decubitus direct current treatment (DDCT) of pressure ulcers: results of a randomized double-blinded placebo controlled study.** *Arch Gerontol Geriatr* 2005**, 41:**261-9**.** |
| 59 | Afshari M, Larijani B, Fadayee M, Ghahary A, Pajouhi M, Bastanhagh MH, Baradar-Jalili R, Vassigh AR, Darvishzadeh F: **Efficacy of topical epidermal growth factor in healing diabetic foot ulcers.** *Therapy* 2005*.* **2:**759-765. |
| 60 | Ahmad ET: **High voltage pulsed galvanic stimulation: effect of treatment durations on healing of chronic pressure ulcers.** *Indian Journal of Physiotherapy and Occupational Therapy* 2008 **2:**1-5. |
| 64 | Alleva R, Tomasetti M, Sartini D, Emanuelli M, Nasole E, Di Donato F, Borghi B, Santarelli L, Neuzil J: **Alpha-Lipoic Acid modulates extracellular matrix and angiogenesis gene expression in non-healing wounds treated with hyperbaric oxygen therapy.** *Molecular Medicine* 2008, **14:** 175-183. |
| 68 | Bhansali A, Venkatesh S, Dutta P, Dhillon MS, Das S, Agrawal A:  **Which is the better option: recombinant human PDGF-BB 0.01% gel or standard wound care, in diabetic neuropathic large plantar ulcers off-loaded by a customized contact cast?** *Diabetes Research and Clinical Practice* 2009. **83:**e13-e16. |
| 69 | Bayram Y, Deveci M, Imirzalioglu N, Soysal Y, Sengezer M: **The cell based dressing with living allogenic keratinocytes in the treatment of foot ulcers: a case study.** *British Journal of Plastic Surgery* 2005, **58:**988-996. |
| 70 | Caravaggi C, Sganzaroli A, Fabbi M, Cavaiani P, Pogliaghi I, Ferraresi R, Capello F, Morabito A: **Nonwindowed nonremovable fiberglass off-loading cast versus removable pneumatic cast (AircastXP Diabetic Walker) in the treatment of neuropathic noninfected plantar ulcers.** *Diabetes Care* 2007, **20:**2577-2578. |
| 73 | Caetano KS, Frade MA, Minatel DG, Santana LA, Enwemeka CS: **Phototherapy improves healing of chronic venous ulcers.** *Photomedicine and Laser Surgery* 2009, **27:**111-118. |
| 77 | Daroczy J: **Quality control in chronic wound management: the role of local Povidone-Iodone (Betadine) therapy.** *Dermatology* 2006, **212(Supp 1):**82-87. |
| 82 | Edwards H, Courtney M, Finlayson K, Shuter P, Lindsay E: **A randomised controlled trial of a community nursing intervention: improved quality of life and healing for clients with chronic leg ulcers.** *Journal of Clinical Nursing* 2009, **18:**1541-1549. |
| 84 | Eccles, N. K. and Hollinworth, H: **A pilot study to determine whether a static magnetic device can promote chronic leg ulcer healing.** *Journal of Wound Care* 2005, **14:**64-67 |
| 104 | Harley J, Harcourt D, Hutchinson B, McLean M, Long M: **A comparative trial of long stretch compression bandaging versus multi-layer compression bandaging in the treatment of chronic venous ulcers.** *Primary Intention* 2004, **12:**6-14. |
| 107 | Jørgensen B, Price P, Andersen KE, Gottrup F, Bech-Thomsen N, Scanlon E, Kirsner R, Rheinen H, Roed-Petersen J, Romanelli M, Jemec G, Leaper DJ, Neumann MH, Veraart J, Coerper S, Agerslev RH, Bendz SH, Larsen JR, Sibbald RG: **The silver-releasing foam dressing, Concreet Foam, promotes faster healing of critically colonised venous leg ulcers: a randomised controlled trial.** *International Wound Journal* 2005, **2:**64-73 |
| 110 | Konig, M., Vansheidt, W., et al. (2005). **Enzymatic versus autolytic debridement of chronic leg ulcers: a prospective randomised trial.** *Journal of Wound Care* 2005*,* **14:**320-323. |
| 124 | Makhsous M, Lin F, Knaus E, Zeigler M, Rowles DM, Gittler M, Bankard J, Chen D: **Promote pressure ulcer healing in individuals with spinal cord injury using an individualised cyclic pressure-relief protocol.** *Advances in Skin and Wound Care* 2009, **22:**514-521. |
| 135 | Ogrin R, Darzins P, Khalil Z: **The use of sensory nerve stimulation and compression bandaging to improve sensory nerve function and healing of chronic venous leg ulcers.** *Current Aging Science* 2009, **2:**72-80. |
| 137 | Polignano R, Bonadeo P, Gasbarro S, Allegra C: **A randomised controlled trial of four-layer compression versus Unna's Boot for venous ulcers.** *Journal of Wound Care* 2004, **13:**21-24. |
| 141 | Purandare H, Supe A: **Immunomodulatory role of *Tinospora cordifolia* as an adjuvant in surgical treatment of diabetic foot ulcers: a prospective randomized controlled trial.** *Indian Journal of Medical Science* 2007, **61:**347-355. |
| 152 | Sert M, Soydas B, Aikimbaev K, Tetiker T: **Effects of iloprost (a prostacyclin analogue) on the endothelial dysfuction and foot ulcers in diabetic patients with peripheral arterial disease.** *International journal of diabetes and metabolism,* 2008, **16:**7-11. |
| 155 | Smeets R, Ulrich D, Unglaub F, Wöltje M, Pallua N: **Effect of oxidised regenerated cellulose/collagen matrix on proteases in wound exudate of patients with chronic venous ulceration.** *International Wound Journal* 2008, **5:**195-203. |
| 158 | Subbanna PK, Margaret Shanti FX, George J, Tharion G, Neelakantan N, Durai S, Chandy SJ, Mathew BS, Suresh R: **Topical phenytoin solution for treating pressure ulcers: a prospective, randomized, double-blind clinical trial.** *Spinal Cord* 2007, **45:**739-743. |
| 159 | Stechmiller JK, Langkamp-Henken B, Childress B, Herrlinger-Garcia KA, Hudgens J, Tian L, Percival SS, Steele R: **Arginine supplementation does not enhance serum nitric oxide levels in elderly nursing home residents with pressure ulcers.** *Biological Research for Nursing* 2005, **6:**289-299. |
| 162 | Taradaj, J. Franek, A. Brzezinska-Wcislo, L. Blaszczak, E. Polak, A: **Randomized trial of medical compression stockings versus two-layer short-stretch bandaging in the management of venous leg ulcers.** *Phlebologie* 2009, **38:**157-163. |
| 163 | Tondi P, Gerardino L, Santoliquido A, Pola R, Gabrielli M, Papaleo P, Gasbarrini A, Pola P, Flore R: **Treatment of ischemic ulcers of the lower limbs with Alprostadil (Prostaglandin E1).** *Dermatological Surgery* 2004, **30:**1113-1117. |
| 164 | Tumino G, Masuelli L, Bei R, Simonelli L, Santoro A, Francipane S: **Topical treatment of chronic venous ulcers with sucralfate: a placebo controlled randomized study.** *International Journal of Molecular Medicine* 2007, **22:**17-23. |
| 165 | Viswanathan V, Pendsey S, Sekar N, Murthy GSR, **Phase III study to evaluate the safety and efficacy of recombinant human epidermal growth factor (REGEN-DTM 150) in healing diabetic foot ulcers.** *Wounds* 2006, **18:**186-196. |
| 179 | Cameron J, Hoffman D, Wilson J, Cherry G: **Comparison of two peri-wound skin protectants in venous leg ulcers: a randomised controlled trial.** *Journal of Wound Care* 2005, **14:**233- 236 |
| 185 | Franek A, Chmielewska D, Brzezinska-Wcislo L, Slezak A, Blaszczak E. **Application of various power densities of ultrasound in the treatment of leg ulcers.** *Journal of Dermatological Treatment* 2004, **15:**379-386. |
| 186 | Janković A, Binić I: **Frequency rhythmic electrical modulation system in the treatment of chronic painful leg ulcers.** *Archives of Dermatological Research* 2008, **300:**377-383. |
| 188 | Martínez-Sánchez G, Al-Dalain SM, Menéndez S, Re L, Giuliani A, Candelario-Jalil E, Alvarez H, Fernández-Montequín JI, León OS: **Therapeutic efficacy of ozone in patients with diabetic foot.** *European Journal of Pharmacology* 2005, **523:**151-161. |
| 191 | Norkus A, Dargis V, Thomsen JK, Harding KG, Ivins N, Serra N, Torres de Castro OG, Galindo A, Andersen KE, Roed-Petersen J, Gottrup F, Blanco JL, de Mena MA, Hauschild A, Moll I, Svensson A, Carter K: **Use of a hydrocapillary dressing in the management of highly exuding ulcers: a comparative study.** *Journal of Wound Care* 2005, **14:**429-432. |
| 194 | Shojaei H., Sokhangoei Y, Soroush ME:  **Low level laser therapy in the treatment of pressure ulcers in spinal cord handicapped veterans living in Tehran.** *Iran Journal of Medical Science* 2008, **33:**44-48. |
| 195 | You WH, Wang P, Li MQ, Zhang Y, Peng YL, Zhang FL: **Therapeutic effects of modified Danggui Sini Decoction on plasma level of advanced glycation end products in patients with Wagner grade 0 diabetic foot: a randomized controlled trial.** *Journal of Chinese Integrative Medicine* 2009, **7:**22-28. |
| 201 | Agrawal RP, Jhajharia A, Mohta N ,Dogra R, Chaudhari V, Nayak KC: **Use of a platelet-derived growth factor gel in chronic diabetic foot ulcers.** *The Diabetic Foot Journal* 2009, **12:**80-88 |
| 202 | Efrati S, Gall N, Bergan J, Fishlev G, Bass A, Berman S, Hamad-Abu R, Feigenzon M, Weissgarten J: **Hyperbaric oxygen, oxidative stress, NO bioavailability and ulcer oxygenation in diabetic patients.** *Undersea and Hyperbaric Medicine* 2009, **36:**1-12. |
| 203 | Quatresooz P, Kharfi M, Paquet P, Vroome V, Cauwenbergh G, Piérard GE: **Healing effect of ketanserin on chronic leg ulcers in patients with diabetes.** *Journal of the European Academy of Dermatology and Venereology* 2006, **20:**277-281 |
| 204 | Tom WL, Peng DH, Allaei A, Hsu D, Hata TR: **The effect of short-contact topical Tretinoin therapy for foot ulcers in patients with diabetes.** *Archives of Dermatology* 2005, **141:**1373-1377. |
| 206 | Duzgun AP, Satir HZ, Ozozan O, Saylam B, Kulah B, Coskun F: **Effect of hyperbaric oxygen therapy on healing of diabetic foot ulcers.** *The Journal of Foot and Ankle Surgery* 2008, **47:**515-519. |
